# Employing the SpyTag-SpyCatcher Reaction for the Modification of Supramolecular Polymers with Functional Proteins

**DOI:** 10.1021/acs.bioconjchem.5c00046

**Published:** 2025-05-14

**Authors:** Fenna W.B. Craenmehr, Alexander Gräwe, Victor A. Veenbrink, Riccardo Bellan, Maarten Merkx, Patricia Y.W. Dankers

**Affiliations:** † Institute for Complex Molecular Systems, 3169 Eindhoven University of Technology, Eindhoven 5600 MB, The Netherlands; ‡ Department of Biomedical Engineering, Laboratory of Chemical Biology, 3169 Eindhoven University of Technology, Eindhoven 5600 MB, The Netherlands; ¥ Department of Chemical Engineering and Chemistry, 3169 Eindhoven University of Technology, Eindhoven 5600 MB, The Netherlands

## Abstract

Supramolecular assemblies hold great potential as biomaterials for several biomedical applications. The modification of supramolecular biomaterials is needed to achieve controlled bioactive functions. Supramolecular ureidopyrimidinone (UPy) monomers have been shown to assemble into long supramolecular polymers that can be functionalized with bioactive peptides and visualized as UPy-fibers. So far, the introduction of biological functionality has been limited to small molecules and peptides. Here, we describe a general method based on SpyTag-SpyCatcher chemistry for conjugating full-length proteins with biologically relevant functions to μm-long UPy fibers via native peptide bond formation, yielding 100% conversion in a 5:95 mol % coassembly of UPy-SpyTag with UPy-glycinamide. The conjugation of monoclonal antibodies is performed using photo-cross-linkable protein G domains. We demonstrate intact fibers and colocalization of antibodies and UPy-fibers using biophysical and imaging methods and achieve recruitment of supramolecular assemblies to the surface of mammalian cells via the EGFR-specific antibody Cetuximab. The approach introduced here represents a robust and widely applicable postassembly modification method that shows promise in the functionalization of future biomaterials.

## Graphical abstract

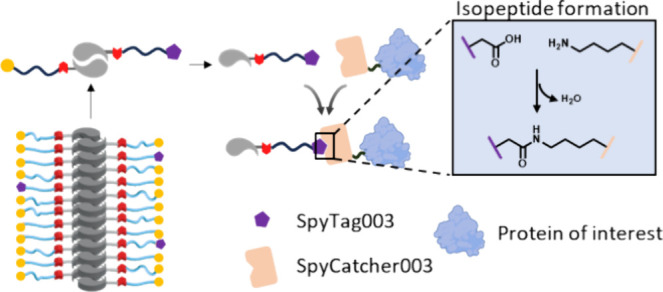

## Introduction

Structurally and functionally well-defined biomaterials have enabled significant progress in therapeutics, diagnostics, and antimicrobial coatings.^1^ Specifically, supramolecular architectures based on non-covalent interactions are useful as they share features with naturally occurring systems, such as dynamic and bioactive properties. The construction of supramolecular architectures with designed function requires a deep understanding of the building blocks, assembly conditions, and parameters.^2^ Supramolecular, dynamic polymers have, among others, shown potential in drug delivery, tissue engineering, antimicrobial materials, and retroviral gene delivery. These functions are often achieved by functionalization of the supramolecular building blocks with peptide motifs.^3–5^ The peptide motifs are often extracted from natural proteins and have advantages such as low molecular weights and ease of production.^6^ When compared to peptide motifs, full-length proteins are known to bring advantages such as higher affinity and higher specificity through a larger molecular interaction interface.^7^

Site-specific and selective functionalization of proteins is challenged by the large number of reactive sites available in a protein, a limitation that is often not present when peptide motifs are used. Some approaches for functionalizing full-length proteins, such as chemical modification of accessible lysine residues with *N*-hydroxysuccinimidyl (NHS) esters,^8,9^ result in heterogeneous populations since lysine residues are abundant on protein surfaces.^10^ As these methods are not site-specific, they reduce the reproducibility and control over the envisioned molecular architecture. To expand the potential applications of supramolecular polymers, the aforementioned limitations must be overcome. Chemical modification of peptide and protein motifs is synthetically accessible through chemoselective ligation and modification strategies.^11–13^ Methods for site-specific labelling of antibodies have been developed and allow a high degree of control over downstream applications.^14,15^ Howarth and coworkers developed the SpyTag/SpyCatcher technology,^16,17^ a chemoselective ligation strategy that allows covalent linkage of two subunits, namely a small peptide SpyTag and a ∼16 kDa protein SpyCatcher, via spontaneous formation of a native peptide bond. The versatile SpyTag/SpyCatcher technology has found applications in various fields, including bacterial protein localization,^18^ the development of dendrimers for T-cell activation,^17^ and the engineering of protein hydrogels.^19^ Despite the many chemoselective ligation strategies available, the relatively large size and complex tertiary structure of proteins, in particular, antibodies, have limited combinations with supramolecular platforms.^20^

Stupp and coworkers utilized native chemical ligation to attach bioactive peptides and fluorescent proteins to self-assembled peptide amphiphile nanofibers, allowing for independent control over self-assembly and bioactivity.^21^ At the same time, Collier and coworkers reported self-assembled peptide-based nanomaterials with multiple different cointegrated proteins. Each protein was fused to a β-tail tag, which assembles with a ß-sheet fibrillizing peptide to form nanofibers with active protein domains.^22^ Unlike supramolecular materials that are functionalized with proteins, proteins themselves have been employed to create supramolecular assemblies.^23,24^

Our group has previously used supramolecular structures based on ureidopyrimidinone (UPy) molecules that are highly controllable in terms of rigidity and architecture. On the nanometer level, UPy molecules are self-complementary and dimerize based on a four-fold hydrogen bonding array.^25^ In water, these hydrogen bonds must be shielded. Therefore, the UPy moiety is shielded by a hydrophobic spacer embedding a urea group, which enhances the dimerization as well as the one-dimensional elongation of the fibers through lateral hydrogen bonding. Further assembly occurs by bundling of the stacks into triplicate fibers. Recently, we coupled functional peptides to UPy fibers, such as recombinant peptides based on human collagen type I, RCPhC1, that contain integrin binding motifs (RGD),^26^ and GFOGER.^27^ Functionalization of fluorescent proteins has previously been achieved via cucurbituril immobilization.^28^ Although the size of the additive increased from peptide to protein, cucurbituril immobilization is a non-covalent ligation strategy.

We have shown that the dynamic behavior of these supramolecular UPy fibers can be controlled by modulating the ratio of self-assembling molecules, allowing for effective incorporation of cell-adhesive functionalities.^29^ In the past, however, UPy assemblies were grafted with RCPhC1 peptides that were dissolved in DMSO, a process that is incompatible with complex folded full-length proteins. Furthermore, the initial UPy-fiber assembly required either a pH switch or elevated temperatures (70 °C), which is unsuited for maintaining protein functionality. Ideally, the protein tertiary structure is not compromised during or after the fiber assembly process.

To elevate the potential of supramolecular UPy assemblies beyond cell adhesion, innovative solutions are required to decorate UPy assemblies and biomaterials with bioactive molecules. There is potentially a strong synergy between supramolecular assemblies and bioactive proteins that confer functions to modulate, e.g., the immune system or cellular responses. IgG antibodies are ideal bioactive proteins as they are well studied, have high affinity toward their targets, such as cell surface receptors, and are readily commercially available.

Here, the concept of utilizing self-assembling molecules to integrate biomolecules^30^ will be translated into a novel platform that enables conjugation of full-length proteins, specifically antibodies, to pre-assembled UPy-fibers. Proteins are covalently bonded to UPy molecules via SpyTag/SpyCatcher technology, forming a native peptide bond (Figure 1A).^31^

**Figure 1:**
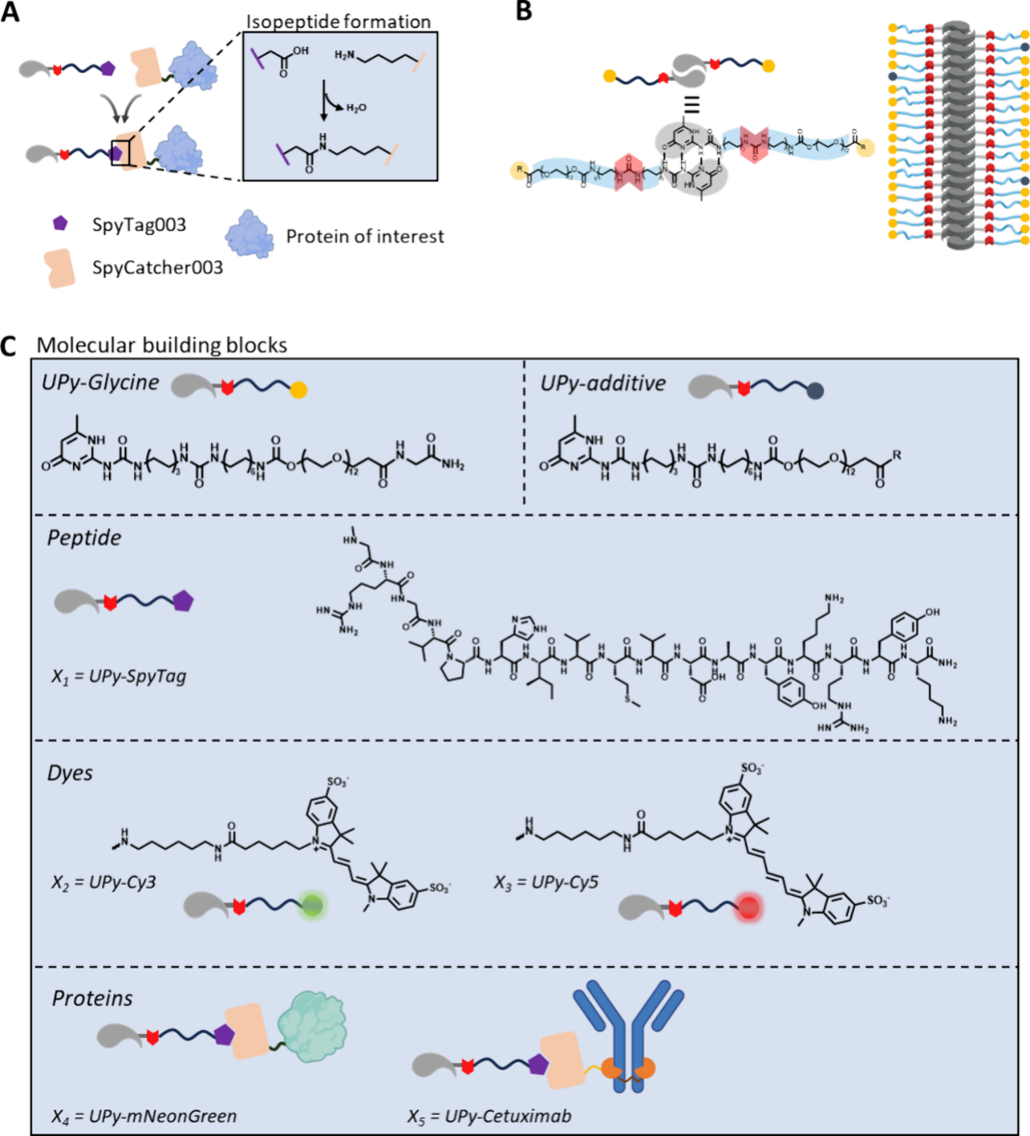
Aim and molecular design of this study. (A) Schematic overview of our aim, in which the SpyTag/SpyCatcher technology is used as a platform for the modification of UPy monomers that can assemble into UPy fibers with a protein of interest. (B) General molecular structure of a UPy molecule that can be functionalized at its R-position (yellow dot). Lateral stacking and assembly results in 1D-fiber formation. (C) Molecular structures of UPy-G and UPy additives that are copolymerized to form fibers. Throughout the study, Sulfo-Cy3 and Sulfo-Cy5 were used as dyes and abbreviated as Cy3 ND Cy5 for better readability.

## Results and Discussion

### Approach

The supramolecular polymers in this study are constructed from monomers that are mixed at different molar ratios. The core structure of the fibers is made of UPy-glycinamide (UPy-G), in which various mol % UPy additives are included (Figure 1B). The hydrophilic tails of the monomers can be functionalized with peptides (such as SpyTag003) or fluorescent moieties (such as Cy3 or Cy5) (Figure 1C). The modularity of this system allows for changes within the UPy fibers by the introduction of various UPy additives. UPy-fibers are formed by a strictly non-covalent assembly procedure using temperature-guided assembly and remain reasonably stable due to specific and directional secondary interactions.^29^ Successful formation of fibers is assessed by various analytical techniques, such as cryo-TEM and TIRF microscopy, to monitor supramolecular assembly.

### Synthesis of UPy-SpyTag and Assembly Behavior

We decided to employ SpyCatcher003 and SpyTag003 as a platform for UPy bioconjugation because this third generation of the Spy-technology was optimized to react with nearly diffusion-limited kinetics of 5.5 ± 0.6 × 10^5^ M^–1^ s^–1^ in aqueous solution.^16^ SpyTag003 (in the following abbreviated as SpyTag) is a small peptide (RGVPHIVMVDAYKRYK, 16 aa, net charge ≈ +3e at neutral pH) without tertiary structure on its own, allowing straightforward attachment to UPy moieties. SpyTag and its derivatives were synthesized via Fmoc-based solid-phase peptide synthesis and purified using preparative HPLC (Figure 2A). Three derivatives of SpyTag were synthesized, with an N-terminal free amine, an OEG_12_-linker, and an UPy-linker, respectively, resulting in 18-31% yield for the peptides. The peptides were analyzed using LC-MS, and the purity (>80%) was determined from the PDA chromatograms at 272 nm.

**Figure 2:**
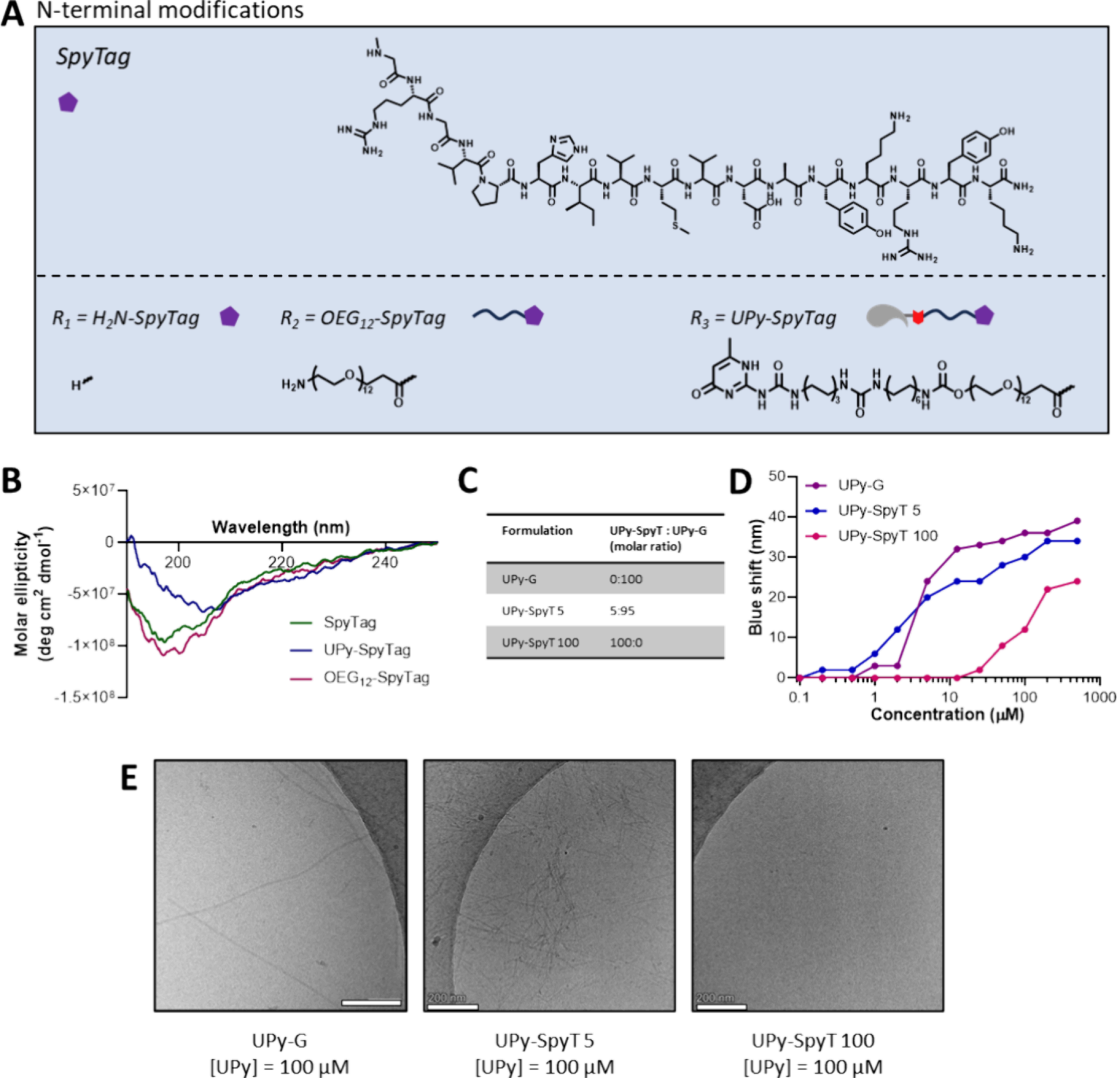
Molecular assembly studies on (UPy-)­SpyTag peptide. (A) Molecular structure of SpyTag peptide and its derivatives. (B) CD spectrum of SpyTag and its N-terminal derivatives, measured at 50 μM in Milli-Q water. (C) Overview of the different ratios of coassemblies used in this study. (D) Nile Red assay of three coassemblies to determine the critical aggregation concentration, measured in Milli-Q water with 250 nM Nile Red. Nile Red is used as the solvatochromic dye. (E) Cryo-TEM images showing μm long fibers for UPy-G and UPy-SpyTag 5, while no fibers are observed for UPy-SpyTag 100. Scale bar = 200 nm.

To investigate the assembly behavior from UPy-SpyTag monomers to fibers, a variety of assembly analysis techniques, including CD spectroscopy, Nile Red analysis, and cryo-TEM, were used. As the addition of the SpyTag peptide sequence to a UPy-molecule might affect the assembly behavior by steric hindrance, UPy-G assemblies were compared with UPy-SpyTag 100 assemblies (containing only UPy-SpyTag) and UPy-SpyTag 5 assemblies (containing 5:95 mol % UPy-SpyTag:UPy-G). First, CD spectroscopy confirmed the SpyTag peptide to be a random coil peptide, as a large negative band is observed near 200 nm (Figure 2B). *N*-terminal modification with an OEG_12_-linker does not affect the CD-spectrum, however, a difference in molar ellipticity is observed for *N*-terminal modification with a UPy-linker, resulting in a less pronounced secondary random coil structure. Next, UPy-SpyTag was further investigated using the Nile Red assay to determine the critical aggregation concentration (CAC) of the different (co)­assemblies (Figure 2C). UPy-G showed a CAC of 2 μM, while a CAC of 20 μM was observed for UPy-SpyTag 100 (Figure 2D). This 10-fold higher CAC may be caused by charge repulsion between UPy-SpyTag monomers, given that the net charge of SpyTag is positive in neutral or slightly acidic conditions**.** The UPy-SpyTag 5 coassembly showed a CAC of 1 μM. Cryo-TEM of 100 μM solutions of (co)­assemblies confirmed fiber formation for UPy-G and UPy-SpyTag 5 (5:95 mol % UPy-SpyTag:UPy-G, Figure 2E). Well-formed fibers of μm length were observed for both conditions, with an increased number of fibers present for UPy-SpyTag 5. The increased fiber density, however, does not result in network formation. No fibers were observed for UPy-SpyTag 100, suggesting that the blue shift observed at 20 μM in the Nile Red assay is caused by random aggregation and not by fiber formation. Therefore, we used UPy-SpyTag 5 for further conjugation studies.

### Covalent Linkage between UPy-SpyTag and SpyCatcher-mNeonGreen

We investigated whether purified proteins that contain SpyCatcher003 (in the following abbreviated to SpyCatcher) can spontaneously form isopeptide bonds with UPy-SpyTag, and how this process may be influenced by the presence of non-functionalized UPy molecules in monomeric or assembled state (Figure 3A). We chose SpyCatcher fused to fluorescent mNeonGreen (mNG) as a proof-of-concept target protein. The fusion protein (SC-mNG) was produced in E. coli BL21 and purified via StrepTactin affinity column (details in the Supporting Information).

**Figure 3:**
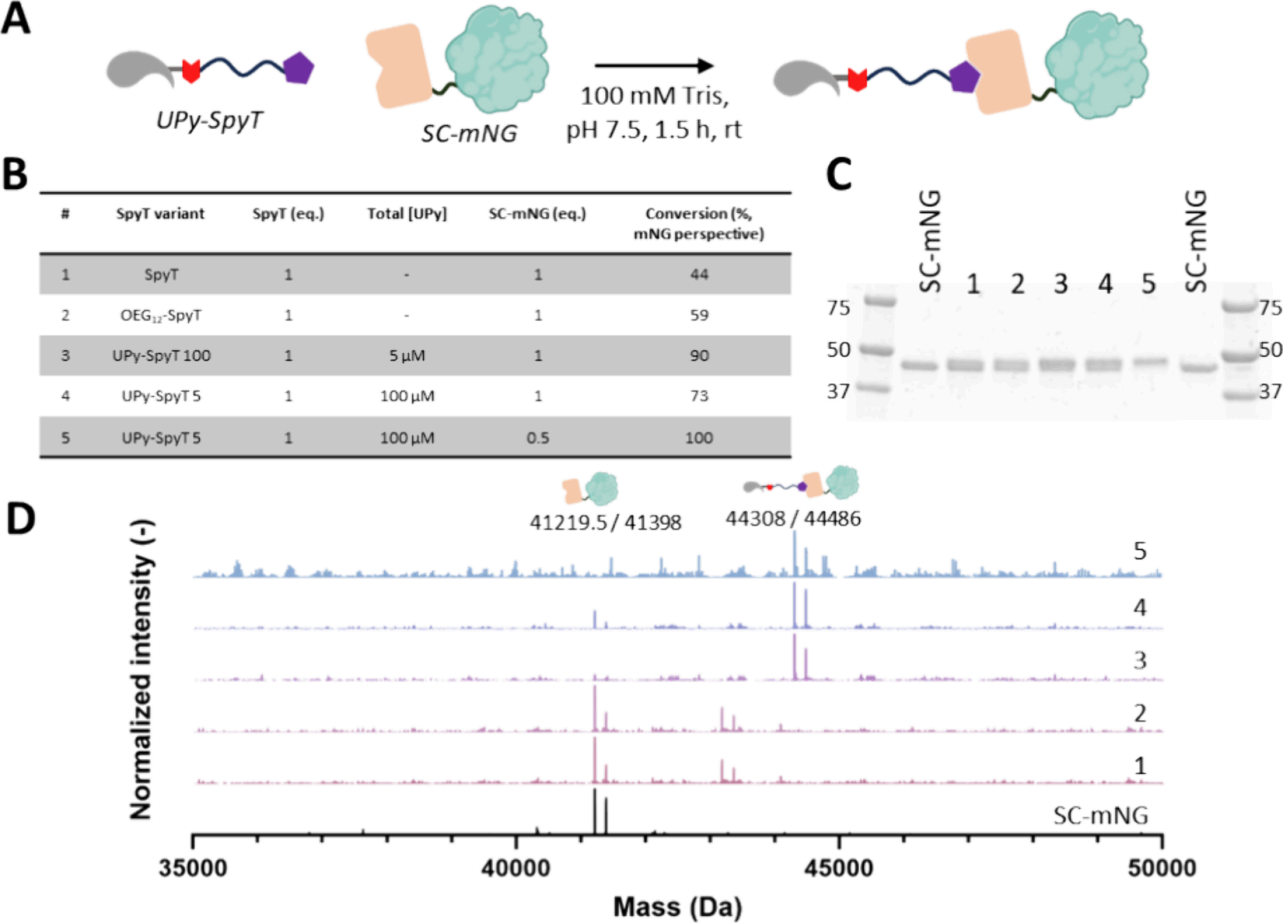
Systematic Study for UPy-SpyTag conjugation with SpyCatcher-mNG. (A) Graphical overview of the performed SpyTag-SpyCatcher reaction. (B) Schematic overview of the investigated reaction conditions, 1 equiv equals 5 μM. Conversion is studied from the SpyCatcher-mNG perspective. (C) Denatured SDS-PAGE analysis, where a complete upwards shift is observed in line 5, indicated increase in molecular mass and thus successful conjugation. Numbers 1–5 correspond to the reaction conditions described in panel B. (D) LC-MS Q-TOF analysis confirming addition of SpyTag variants to SpyCatcher-mNG. Conversions are determined from the corresponding mass spectra. Numbers 1-5 correspond to the reaction conditions described in panel (B).

Product formation under different conditions was assessed via mass spectrometry (LC-MS Q-ToF) and denaturing SDS-PAGE. The reaction partners were mixed at chosen ratios (Figure 3B) and incubated at room temperature (24 °C) for 1.5 h in 100 mM Tris buffer at pH 7.5.

The isopeptide bond formation (conversion) between SpyTag peptide and SpyCatcher-mNeonGreen (SC-mNG) was defined as the ratio of unreacted SC-mNG versus reacted SC-mNG. The conversion between synthesized H_2_N-SpyTag peptide and SC-mNG was 44% when performed at 5 μM scale (Figure 3B), which is lower than the conversion found in the work of Keeble et al.^16^ The efficiency improved to 59% when the SpyTag peptide was *N*-terminally modified with the peptide of OEG_12_. Interestingly, a high conversion of 90% was observed when 5 μM of SC-mNG and UPy-SpyTag were used without the presence of additional UPy-G coassembly moieties. This indicated that the reaction was more efficient in the absence of supramolecular assemblies (as shown in Figure 2E). The SpyTag/SpyCatcher reaction was subsequently tested on a coassembled fiber (UPy-SpyTag 5), resulting in 73% conversion (Figure 3B). This suggested that the isopeptide bond formation was critically dependent on the ratio between UPy-G and UPy-SpyTag and the molar ratio of SpyTag and SpyCatcher. This was confirmed through further investigations, ultimately arriving at a ratio that allowed full conversion of free SpyCatcher to product, which is at a 2 M excess of SpyTag over SpyCatcher and a UPy-SpyTag coassembly (UPy-SpyTag 5, Figure 3A). This result was confirmed by SDS-PAGE, as all free SpyCatcher-mNG had reacted in this condition (lane 5), while a mixture of reacted and free protein was observed for other conditions (lanes 1–4, Figure 3C). Using LC-MS Q-TOF, a mass increase of 3091 Da was observed; this corresponds to the addition of UPy-SpyTag to SpyCatcher-mNG (Figure 3D, lane 5).

TIRF and cryo-TEM were used to visually verify the success of the decoration of UPy-fibers with mNeonGreen (Figure 4). UPy-fibers were pre-aged to μm length, and the stability of the fibers was confirmed using both cryo-TEM and TIRF. This demonstrates that the right assembly conditions and protein ligation technology allow full-length proteins to be attached to UPy assemblies. The fluorescent properties of mNeonGreen (max λ_ex_ = 506 nm, max λ_em_ = 517 nm) allowed us to verify colocalization with Cy5-labeled UPy fibers in TIRF. UPy-SpyTag/SpyCatcher-mNG fibers showed fluorescence in both the green and the red channels (Figure 4A); moreover, mNG and Cy5 appeared to colocalize within the fibers (Figure 4B). Additional analysis by cryo-TEM revealed μm-long fibers (Figure 4C) that are similar to UPy-SpyTag 5 fibers before protein conjugation (Figure 2E).

**Figure 4:**
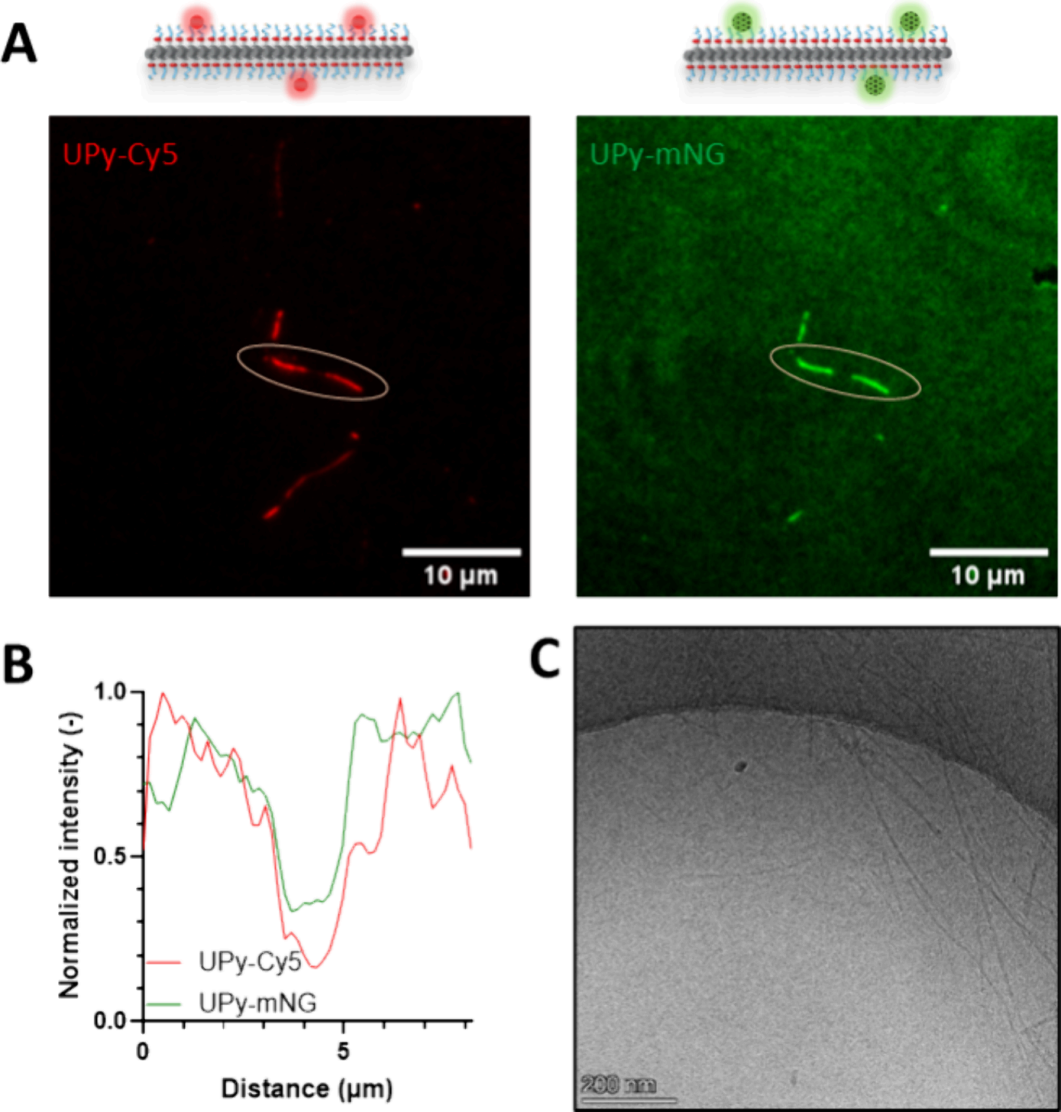
Microscopic analysis of UPy-mNG assemblies. (A) TIRF analysis of UPy-G fibers with UPy-Cy5 (left) and UPy-mNG (right) as molecular additives. Scale bar = 10 μm. (B) Intensity analysis revealed colocalization of UPy-Cy5 and UPy-mNG among the fiber. The cross section is taken as indicated in panel (A). (C) Cryo-TEM analysis after UPy-SpyTag SpyCatcher-mNG conjugation. Scale bar = 200 nm.

Biophysical and optical characterization of the separate conjugation and assembly steps revealed successful fiber formation (μm length) of UPy assemblies containing up to 5 mol % UPy-SpyTag. This demonstrates that the SpyTag on the fiber did not lose its functionality and was able to bind and form an isopeptide bond with an mNeonGreen-SpyCatcher fusion protein.

### Modification of UPy-SpyTag with Functional Antibodies

The final goal was to decorate UPy fibers with full-length antibodies, an endeavor that would allow precise targeting of UPy-based materials to biologically relevant molecules such as cell surface receptors.^27^ Linkage of antibodies to UPy-SpyTag fibers requires the functionalization of the antibodies with SpyCatcher. In the past, straightforward antibody functionalization with proteins using the LASIC technology was demonstrated.^14^ This technique relies on a protein G (pG) variant that can be covalently linked to the Fc part of most IgG antibodies. This linkage occurs via photoconjugation between the unnatural amino acid para-benzoylphenylalanine (pBPA) incorporated in protein G and a conserved methionine in the antibody Fc part. A dimeric version of protein G, pG_2_, was used to increase the efficiency of photo-cross-linking and ensure 1:1 conjugation (Figure 5A).^32^ Here, a two-step protocol was envisioned to create a UPy-functionalized antibody, wherein the SpyTag-SpyCatcher ligation and LASIC technology are combined to create a UPy-SpyTag-SpyCatcher-pG_2_-antibody complex.

**Figure 5:**
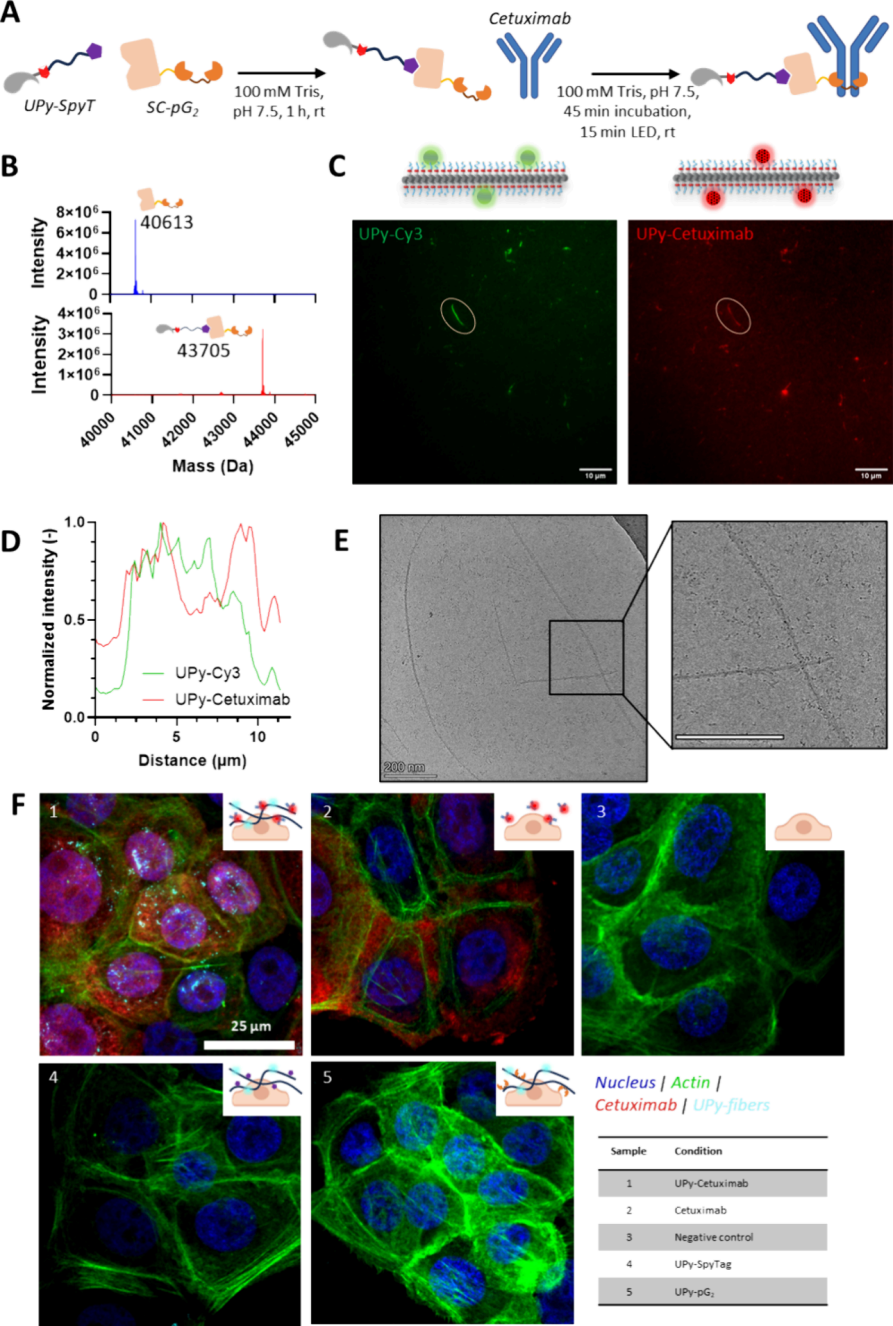
Functionalization of UPy-fibers with functional antibodies. (A) Conceptual overview of conjugation methods to create the UPy-antibody construct. The conjugation in buffer is followed by a final UV-light step to covalently cross-link pG_2_ and antibody. (B) LC-MS Q-TOF analysis of SpyCatcher-pG_2_ construct before (upper) and after (lower) functionalization with UPy-SpyTag. (C) TIRF analysis of UPy-G fibers with UPy-Cy3 (left) and UPy-Cetuximab­(Alexa-647, right) as molecular additives. Scale bar = 10 μm. (D) Intensity analysis revealed colocalization of UPy-Cy3 and UPy-Cetuximab among the fiber. (E) Cryo-TEM analysis of total UPy-antibody complex. Antibodies can be seen as small black dots in the inset. Scale bar = 200 nm (F) Confocal image indicating BxPC3 cells with different UPy assemblies for labeling. UPy assemblies only appear on the image when functionalized with Cetuximab, recognizing EGFR on the cell membrane (UPy-fibers in white, nucleus in blue, actin-cytoskeleton in green, Cetuximab-Alexa647 in red). Scale bar = 25 μm.

The construct pG_2_-SpyCatcher was expressed in E. coli BL21 (DE3) in the presence of pBPA and purified via affinity tags, resulting in pure protein (Figure S1). When mixed with preassembled UPy-SpyTag 5 fibers, pG_2_-SpyCatcher was efficiently conjugated, as confirmed through LC-MS Q-ToF analysis. The conjugation reaction between pG_2_-SpyCatcher and UPy-SpyTag was more efficient than the SpyCatcher-mNG conjugation for all tested conditions, reaching full conversion for SpyTag, UPy-SpyTag, and a UPy-SpyTag coassembly (5:95 mol % UPy-SpyTag:UPy-G, Figure 5B, Figure S2). The protein domain fused to SpyCatcher appeared to have an influence on the efficiency of the SpyCatcher/SpyTag reaction, as mNG performed worse than pG_2_ in multiple conditions. We speculate this is due to (un)­favorable interactions of the attached domain with the other components present in solution. For example, the SpyCatcher-mNG used here contains two cysteines and has a theoretical pI of 5.25, in contrast to SpyCatcher-pG2 with no cysteines and a pI of 4.65.

The successful conjugation of pG_2_ to the coassemblies allowed for decorating supramolecular UPy fibers with full-length antibodies. The anti-EGFR IgG Cetuximab was used as a model antibody due to its high clinical relevance and well-described binding to mammalian cells.^33^ pG_2_-decorated UPy fibers were mixed with Alexa647-labelled Cetuximab, and UV light was applied to achieve covalent coupling of pG_2_ and antibody.^34^ An SDS-PAGE showed that about 50% of the antibody was successfully conjugated to pG_2_ (Figure S3). The LASIC process can potentially be optimized further by changing the reaction conditions and molar ratio of pG_2_ and antibody parts, provided that the UPy molecules do not inhibit pG_2_ binding or the photoreaction. The method is not limited to Cetuximab and works similarly well with Nivolumab (Figure S3).

TIRF experiments on the complete assemblies showed an overlap of the antibody label (Alexa647) with the UPy label (Cy3) (Figure 5C,D). The assemblies contained 94:5:1 mol % UPy-G:UPy-SpyTag:UPy-Cy3, on which both the SpyCatcher-pG_2_ and Cetuximab conjugation were performed, yielding an estimated ratio of 94:2.5:2.5:1 mol % UPy-G:UPy-pG2:UPy-Cetuximab:UPy-Cy3 based on complete SpyTag-SpyCatcher conjugation and 50% antibody functionalization. TIRF analysis revealed intensity differences between fibers and the background can be seen. Quantified fiber analysis revealed overlap for Cy5 and Alexa647, indicating that UPy-antibody conjugates are incorporated into UPy-G fibers. Although the colocalization is less abundant in comparison to the previous example with mNG, the incorporation of UPy-functionalized Cetuximab is further confirmed by cryo-TEM, showing colocalization of antibodies and well-formed μm-long UPy fibers (Figure 5E). Antibodies are observed as small black dots in the sample, both attached to UPy fibers and also present in solution, as the Cetuximab conjugation was 50% effective.

The binding of Cetuximab-functionalized UPy-fibers to cells was examined by using BxPC3 cells. BxPC3 cells were chosen due to their overexpression of EGFR, the target of Cetuximab, on their cell membranes. Cetuximab retained its functionality after conjugation to the supramolecular polymers, as indicated by the red color in both the UPy-Cetuximab and Cetuximab-only samples. UPy-fibers were specifically attached to the surface of BxPC3 cells only if the fibers were conjugated to Cetuximab (Figure 5F1). The antibody-only control (Figure 5F2) did show spreading of the Cetuximab signal over the whole cell. For UPy-fibers without antibody, the UPy channel did not show the same level of binding, and the UPy-fibers did not cluster at the cell surface (UPy-SpyTag, Figure 5F4 and UPy-pG_2_, Figure 5F5). These results demonstrated that the attachment of supramolecular polymers to the cell surface is mediated by antibody binding.

## Conclusions

We developed a tool in which supramolecular systems can be combined with biological functions. Here, we combined the versatility of UPy chemistry with two proteins, with promising results suggesting that a similar strategy can be applied to other supramolecular systems and other proteins. We characterized the structure and molecular assembly of protein-decorated UPy fibers in detail with biophysical and microscopic techniques. We demonstrated efficient conjugation of functional antibodies to UPy assemblies and target EGFR on the surface of mammalian cells with Cetuximab-functionalized UPy assemblies. This recruitment may allow the induction of a downstream cascade in the targeted cell in the future, provided that additional functional biomolecules are added to the UPy-architecture. The options for reliable protein-ligation systems have recently been expanded, for example, with the DogTag/DogCatcher system, which is orthogonal to the SpyTag/SpyCatcher technology.^35^ In principle, our technology makes it possible to create hybrid fibers that combine supramolecular chemistry with engineered proteins, which can form hydrogels with tunable viscoelasticity.^36^ Upon incorporation of a small amount of cross-linker (a bifunctional UPy molecule) to a UPy-glycinamide (UPy-G) solution, hydrogels with tunable mechanics were created as a cell adhesion matrix. Variations in the ratio of the monofunctional UPy-G and the bifunctional UPy allowed to investigate how matrix stiffness influences cell behavior.^29,37,38^ Further studies will elucidate to what extent large proteins interfere with the UPy fiber formation process or the stability of the fibers over time. With this study, we provide a novel path to achieve bioactive hybrid materials for cell fate triggering, controlled therapeutic release, and personalized healthcare.

## Material and Methods

### Materials

All chemicals and solvents were purchased from commercial sources (VWR, Biosolve) and used as received unless stated otherwise. The synthesis of UPy-G, UPy-Cy3, and UPy-Cy5 was described previously.^29^ UPy-COOH synthon was synthesized as previously described.^39^

### Synthetic Procedures

#### Peptide Synthesis

The SpyTag peptides were synthesized on a 50 μmol scale on a Rink amide MBHA resin (Novabiochem, 0.52 mmol/g loading) via Fmoc-based solid phase peptide synthesis. The resin was swollen for ten min in DMF (Biosolve). Fmoc-deprotection was performed twice per cycle with 20% v/v piperidine in *N*,*N*-dimethylformamide (DMF) for five min, followed by 3 × 30 s washing with DMF. Fmoc-protected amino acids (5 equiv) were mixed with 4.5 equiv of HBTU (0.38M in DMF) and 15 equiv of DIPEA, and coupled to the resin for 30 min under continuous agitation. Afterwards, the resin was washed 3 × 30 s with DMF. The deprotection-washing-coupling-washing cycle was repeated for each amino acid.

*N*-Terminal peptide modification with UPy-C_6_–U-C_12_–Ur-OEG_12_-C_2_–COOH was performed on the resin. UPy-C_6_–U-C_12_–Ur-OEG_12_-C_2_–COOH (2 equiv) was dissolved in DMF (100 mg/mL) before the addition of HATU (4 equiv) and DIPEA (16 equiv). The mixture was of UPy-C_6_–U-C_12_–Ur-OEG_12_-C_2_–COOH, and HATU and DIPEA in DMF were preactivated for 30 min before addition to the resin. The reaction was left overnight with continuous agitation.

*N*-Terminal peptide modification with t-Boc-N-amido-PEG12-acid was performed on the resin following the protocol of a natural amino acid. t-Boc-N-amido-PEG12-acid (5 equiv) was mixed with 4.5 equiv HBTU (0.38M in DMF) and 15 equiv of DIPEA, and coupled to the resin for 30 min under continuous agitation.

After the synthesis, resins were washed 3× with DMF, 3× with DCM, and dried under reduced pressure. Removal of protecting groups and cleavage of the resin was performed by incubation in a mixture of trifluoracetic acid (TFA), H_2_O, and triisopropylsilane (TIS) (96.5:2.5:1) for 2 h with continuous agitation, followed by precipitation in excess of ice-cold diethyl ether (Et_2_O), dried under compressed air, dissolved in Milli-Q and lyophilized to obtain the crude peptide.

All peptides were purified using preparative HP-LC. This was performed using a Gemini S4 110A 150 mm × 21.20 mm column using ultrapure water with 0.1% formic acid (FA) and acetonitrile with 0.1% FA with various gradients. Correct mass and purity of peptides were identified using analytical liquid chromatography coupled with mass spectrometry (LC-MS), performed on a C4 Jupiter SuC4300A 150 × 2.0 mm column using ultrapure water with 0.1% FA and acetonitrile with 0.1% FA. In general, a gradient of 5% to 100% acetonitrile over 10 min was used, connected to a Thermo Fischer LCQ fleet ion trap mass spectrometer. The purity of the samples was assessed by using a UV detector at 272 nm.

##### SpyTag

20 μmol scale, 7 mg, 18% yield. 7 LC-MS (ESI): for C_88_H_143_N_27_O_20_S, molecular weight calc. 1931.34 g/mol, observed retention time 2.45 min, [M+3H]^3+^: 644.82, [M+4H]^4+^: 484.08, [M+5H]^5+^: 387.58. Analysis by LC-MS (UV, 272 nm) indicated >95% purity.

##### G-SpyTag

20 μmol scale, 6.5 mg, 16% yield. LC-MS (ESI): for C_90_H_146_N_28_O_21_S, molecular weight calc. 1988.39 g/mol, observed retention time 2.36 min, [M+3H]^3+^: 663.92, [M+4H]^4+^: 498.33, [M+5H]^5+^: 399.17. Analysis by LC-MS (UV, 272 nm) indicated >95% purity.

##### OEG-SpyTag

10 μmol scale, 8 mg, 31% yield. LC-MS (ESI): for C_117_H_199_N_29_O_34_S, molecular weight calc. 2588.11 g/mol, observed retention time 2.59 min, [M+3H]^3+^: 863.67, [M+4H]^4+^: 648.17, [M+5H]^5+^: 518.92, [M+6H]^6+^: 432.83. Analysis by LC-MS (UV, 272 nm) indicated >80% purity.

##### UPy-SpyTag

20 μmol scale, 13.8 mg, 22% yield. LC-MS (ESI): for C_143_H_243_N_35_O_39_S, molecular weight calc. 3108.78 g/mol, observed retention time 3.18 min, [M+4H]^4+^: 778.43, [M+5H]^5+^: 623.17, [M+6H]^6+^: 519.67. Analysis by LC-MS (UV, 272 nm) indicated >95% purity.

#### Protein Expression & Purification

Proteins containing SpyTag003 or SpyCatcher003 domains were expressed in E. coli BL21 (DE3) cells. After the cells were transformed with corresponding expression plasmids (a pFLinkC-XE version with Kan^r^ instead of Amp^r^),^40^ the cells were grown in LB medium supplemented with 50 μg/mL kanamycin at 37 °C at 180 rpm in a shaking incubator. Large cultures (0.5–2 L) in 2 or 5 L baffled flasks were inoculated with corresponding overnight cultures and induced with IPTG at OD_600_ 0.6–0.8. Proteins were expressed overnight at 20 °C. Harvested cell pellets were lysed with BugBuster reagent (Novagen) supplemented with Benzonase (Merck), and proteins were purified with Strep-Tactin XT (iba) using gravity flow columns. Proteins were eluted in buffer E (150 mM NaCl, 100 mM Tris-Cl pH = 8, 50 mM biotin). The purity of the proteins was confirmed by reducing SDS-PAGE. The protein concentrations were calculated using A_280_ Nanodrop measurements, and molar concentrations were calculated using the theoretical extinction coefficients based on the protein sequences. Aliquots were flash frozen in liquid N_2_ and stored at −80 °C until further use.

#### SpyCatcher-SpyTag Reaction (with SpyCatcher-mNeonGreen)

UPy assemblies were made as described in the Noncovalent Assembly Procedure for UPy Molecules section. For H_2_N-SpyTag and OEG_12_-SpyTag, the same heating, vortexing, and cooling protocol was followed. SpyTag variants (1 equiv, 5 μM SpyTag) were mixed with SpyCatcher-mNeonGreen (5 μM) and incubated for 1.5 h at 22 °C. For UPy-SpyTag 5 (5:95 mol % UPy-SpyTag:UPy-G), a 2:1 ratio of UPy-SpyTag to SpyCatcher-mNeonGreen is also investigated.

#### SpyCatcher-SpyTag Reaction (with pG_2_-SpyCatcher) and Antibody Photoconjugation (LASIC)^14^

UPy fibers with UPy-SpyTag at 2.5 μM were mixed with pG_2_-SpyCatcher (final concentration 1.5 μM) and incubated for 1 h at 22 °C. Following assembly of UPy-SpyTag and pG_2_-SpyCatcher, the antibody was mixed with the assembly reaction in a 2:1 pG_2_:antibody molar ratio (total volume 50 μL) and incubated for 45 min at 22 °C. Afterward, the mixture was irradiated with UV light (Thorlabs M365LP1 with a Thorlabs LEDD1B T-Cube LED Driver) for 15 min. Successful conjugation was checked via SDS-PAGE (Figure S3). No further purification of the antibody-UPy conjugates was performed. The conjugates were stored at 4 °C until use.

### Methods

#### Noncovalent Assembly Procedure for UPy Molecules

Solid UPy powders were weighed into 1.5 mL of clean, separate glass vials. Then, the solids were dissolved in an adequate amount of 1x PBS (pH 7.4). The samples were heated for 15 min at 70 °C. After heating, the samples were vortexed for 20 s and cooled to room temperature. The UPy-molecules were diluted (and/or coassembled) to the desired concentration in 1× PBS, heated again for 15 min at 70 °C, vortexed for 20 s, and cooled to room temperature. After the reaction, the samples were stored on the bench (at room temperature, without agitation) for 1 week before further analysis using TIRF and cryo-TEM.

#### LC-MS Iontrap Analysis

Liquid chromatography coupled with mass-spectrometry (LC-MS) was performed on a C4 Jupiter SuC4300A 150 x 2.0 mm column using ultrapure water with 0.1% formic acid (FA) and acetonitrile with 0.1% FA, in general with a gradient of 5% to 100% acetonitrile over 10 min, connected to a Thermo Fisher LCQ Fleet Ion Trap Mass Spectrometer. The purity of the samples was assessed using a UV detector at 272 nm.

#### LC-MS Q-TOF Analysis

Liquid chromatography coupled with mass-spectrometry (LC-MS) was performed on a Polaris 3 C8-A 100 × 2.0 mm column using ultrapure water with 0.1% formic acid (FA) and acetonitrile with 0.1% FA, in general with a gradient of 15 to 75% acetonitrile over 8 min, connected to a Xevo G2 Q-TOF Mass Spectrometer.

#### Circular Dichroism

CD spectra were measured on a JASCO J-815 CD Spectrometer equipped with a Peltier temperature controller. Measurements were performed at 20 °C in Quartz cuvettes with a path length of 1 cm and a UPy concentration of 50 μM. A scanning rate of 100 nm/min, a bandwidth for monitoring of 1.0 nm, a response time of 1 s, a data pitch of 0.1 nm, triple accumulation, and a scanning range of 250–190 nm were employed.

#### Nile Red

Fluorescence spectroscopy with Nile Red was measured on a Cary Eclipse fluorescence spectrophotometer. Measurements were performed at 20 °C. The sample was excited at 550 ± 10 nm, and the emission was recorded at 565–800 nm with fast scan control, medium PMT detector voltage, averaging 5 scansions.

#### Cryo-TEM

Vitrified thin films for cryo-TEM analysis were prepared using an automated vitrification robot (FEI Vitrobot Mark IV) by plunge vitrification in liquid ethane. Before vitrification, a 200-mesh copper grid covered with a Quantifoil R 2/2 holey carbon film (Quantifoil Micro Tools GmbH) was surface plasma treated for 40 s using a Cressington 208 carbon coater. CryoTEM imaging was carried out on the Glacios (Thermo Fisher), equipped with a field emission gun (X-FEG), Ceta 16M camera, and a Falcon 4i direct electron detector. The microscope was operated at 200 kV acceleration voltage in bright-field TEM at a nominal magnification of 6.500× and a dose rate of 2 e–/Å^2^·s, or at 24.000× magnification and a dose rate of 4 e–/Å^2^·s, both with a 1 s image acquisition time.

#### TIRF

Total internal reflection fluorescence (TIRF) microscopy was performed with a Nikon N-STORM system. Fluorescence was collected by means of a Nikon ×100, 1.4NA oil immersion objective and passed through a quad-band-pass dichroic filter (97335 Nikon). Images were recorded with an EMCCD camera (iXon3, Andor, pixel size 0.16 μm) and analyzed using FIJI. Cy5 and Alexa-647 were excited using a 647 nm laser, and Cy3 and mNeonGreen were excited using a 561 nm laser.

#### Cell Culture

BxPC-3 cells (ATCC, CRL-1687) were cultured in RPMI-1640 medium (ATCC) supplemented with 10% (v/v) fetal bovine serum (Invitrogen) and 1 v/v% penicillin–streptomycin (Invitrogen). Cells were kept in an incubator at 37 °C with 5% CO_2_. The medium was replaced every 2–3 days. Cells were passaged using trypsin/EDTA (Invitrogen) once they reached 90% confluency. Cells with passage numbers between 8 and 10 were used for experiments.

#### Immunofluorescence Staining and Confocal Microscopy

Cells were seeded in μ-Slide 15-well 3D slides (Ibidi) at a density of 1800 cells/well. Cells were allowed to adhere and recover for 24 h in an incubator at 37 °C with 5% CO_2_. Following this, the cells were washed twice with PBS and fixed using 3.7 v/v% formaldehyde in PBS for 10 min at 37 °C. Next, the cells were washed twice with PBS and blocked using 1 w/v% BSA for 30 min at room temperature. The cells were washed twice with PBS and stained using Cetuximab-647, UPy-Cetuximab-647, UPy-pG_2_, or UPy-SpyTag in PBS for 1 h in the dark; all conditions with UPy contained UPy-Cy5 for visualization. Following this, the cells were washed once with PBS and stained with phalloidin-488 (1:200) and DAPI (1:200) in PBS. Finally, cells were washed once with PBS and mounted in Mowiol. Samples were imaged on a LEICA TCS SP8 inverted confocal microscope using an HC PL APO 40×/0.95 dry objective.

## Supplementary Material



## Notes

Protein sequences and supplementary figures on protein purification (pG_2_-SpyCatcher), LC-MS Q-TOF analysis of SpyTag–pG_2_-SpyCatcher conjugations, SDS-PAGE of antibody–pG_2_-SpyCatcher conjugations, and additional confocal images of cell studies (PDF)
