# Supplementary material for: Employing the SpyTag-SpyCatcher Reaction for the Modification of Supramolecular Polymers with Functional Proteins
Source: Bioconjug Chem. 2025 May 14;36(6):1197–207. doi: 10.1021/acs.bioconjchem.5c00046 (PMC12186833; doi:10.1021/acs.bioconjchem.5c00046)
Supplement: Supplementary file 1 [file bc5c00046_si_001.pdf]

# Supporting Information

**Employing the SpyTag-SpyCatcher reaction for the modification of supramolecular polymers with functional proteins**

Fenna W.B. Craenmehr[†,‡,§], Alexander Gräwe[†,‡,§], Victor A. Veenbrink[†,‡], Riccardo Bellan[†,‡], Maarten Merkx[†,‡] and Patricia Y.W. Dankers[†,‡,¥,#]

[†] Institute for Complex Molecular Systems, Eindhoven University of Technology, The Netherlands
[‡] Department of Biomedical Engineering, Laboratory of Chemical Biology, Eindhoven University of Technology, The Netherlands
[¥] Department of Chemical Engineering and Chemistry, Eindhoven University of Technology, The Netherlands

## Supporting figures

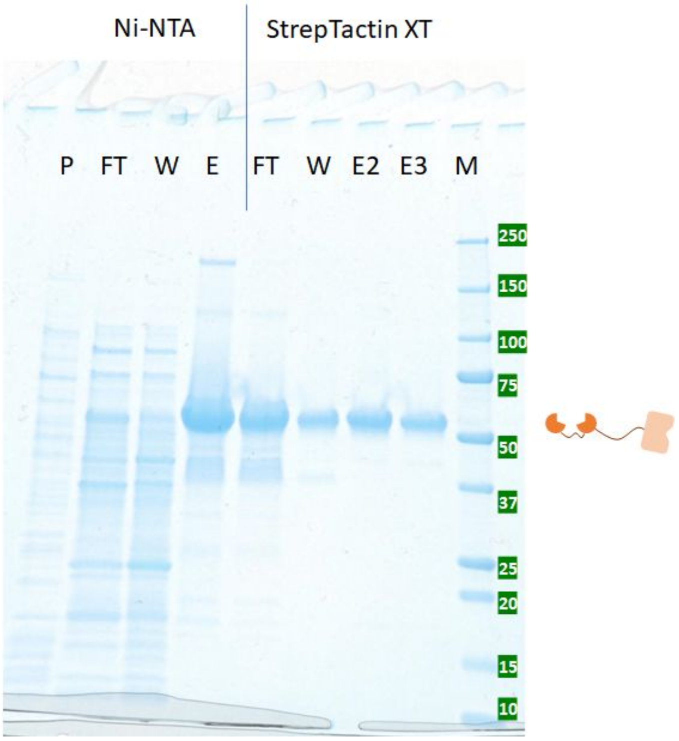


**Fig. S1 |** Purification of $pG_2$-SpyCatcher. Denaturing SDS-PAGE of $pG_2$-SpyCatcher; two step purification (Ni-NTA column and StrepTactin XT column). P: pellet fraction, FT: Flowthrough fraction, W: wash fraction, E2 / E3: Elution fractions. M: Marker.

**A**

| # | SpyT variant | SpyT (eq.) | Total [UPy] | SC-mNG (eq.) | Conversion (%, $pG_2$ perspective) |
|---|---|---|---|---|---|
| 1 | SpyT | 2 | - | 1 | 100 |
| 2 | UPy-SpyT 100 | 2 | 5 µM | 1 | 100 |
| 3 | UPy-SpyT 5 | 1 | 100 µM | 1 | 100 |

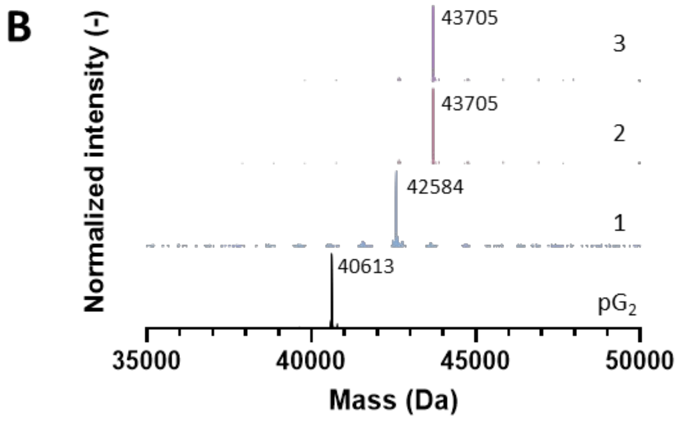


**Fig. S2 |** LC-MS Q-TOF analysis of SpyTag – $pG_2$-SpyCatcher conjugations using different reaction conditions (1-3).

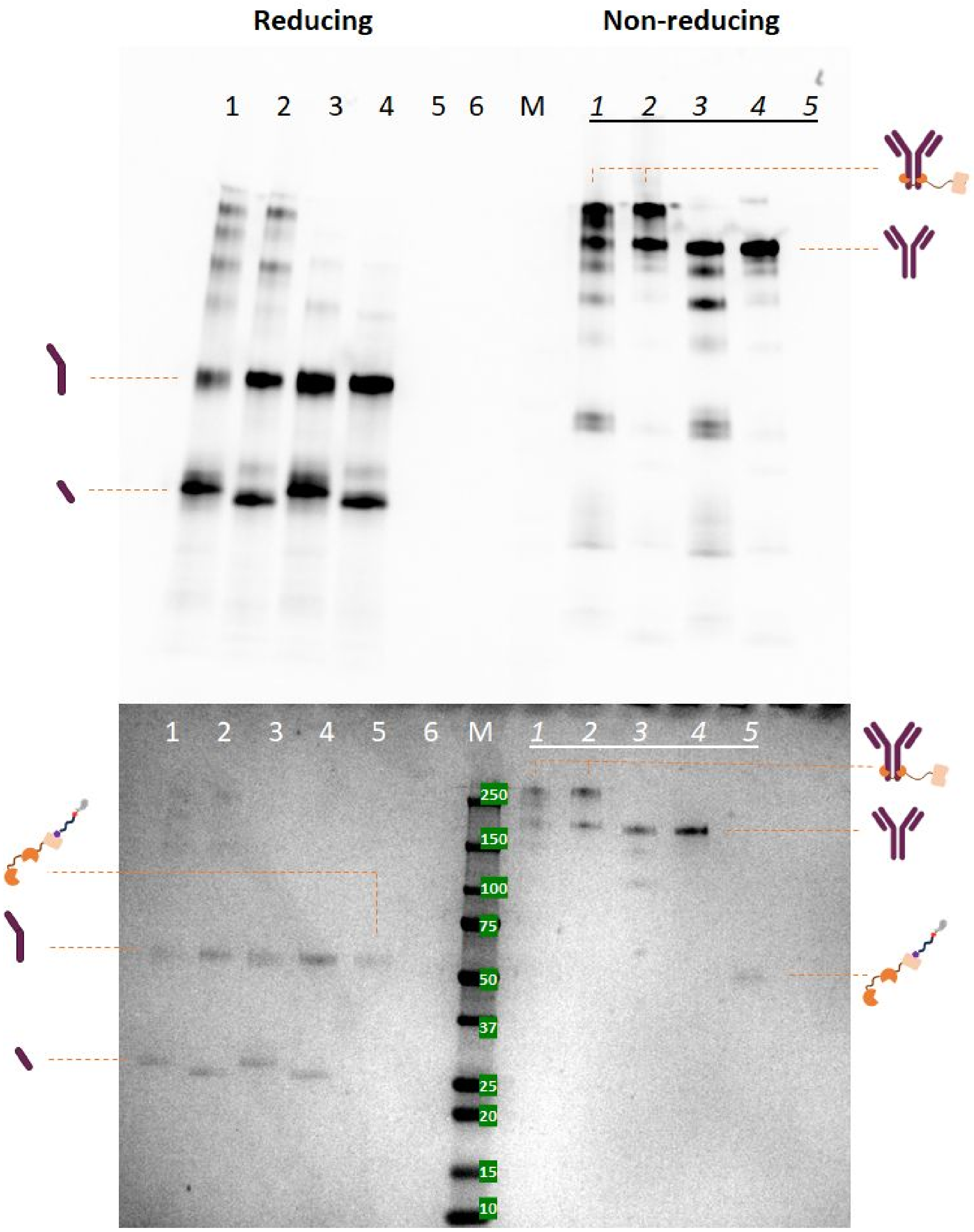


**Fig. S3 |** SDS-PAGE of antibody/pG$_2$-SpyCatcher conjugations, in presence of DTT (reducing) or without DTT added (non-reducing, italic and underlined), after UV irradiation. The top part was imaged via fluorescent mode, exciting the Alexa-647 dye on the antibodies. The lower part was imaged via camera after Coomassie staining. 1: Cetuximab & pG$_2$-SpyCatcher, 2: Nivolumab & pG$_2$-SpyCatcher, 3: Cetuximab, 4: Nivolumab, 5: UPy-SpyTag/pG$_2$-SpyCatcher, 6: UPy-SpyTag (too small to be seen). Comics denote the expected molecules seen in the lanes (full-length antibodies, conjugates, heavy chains, light chains).

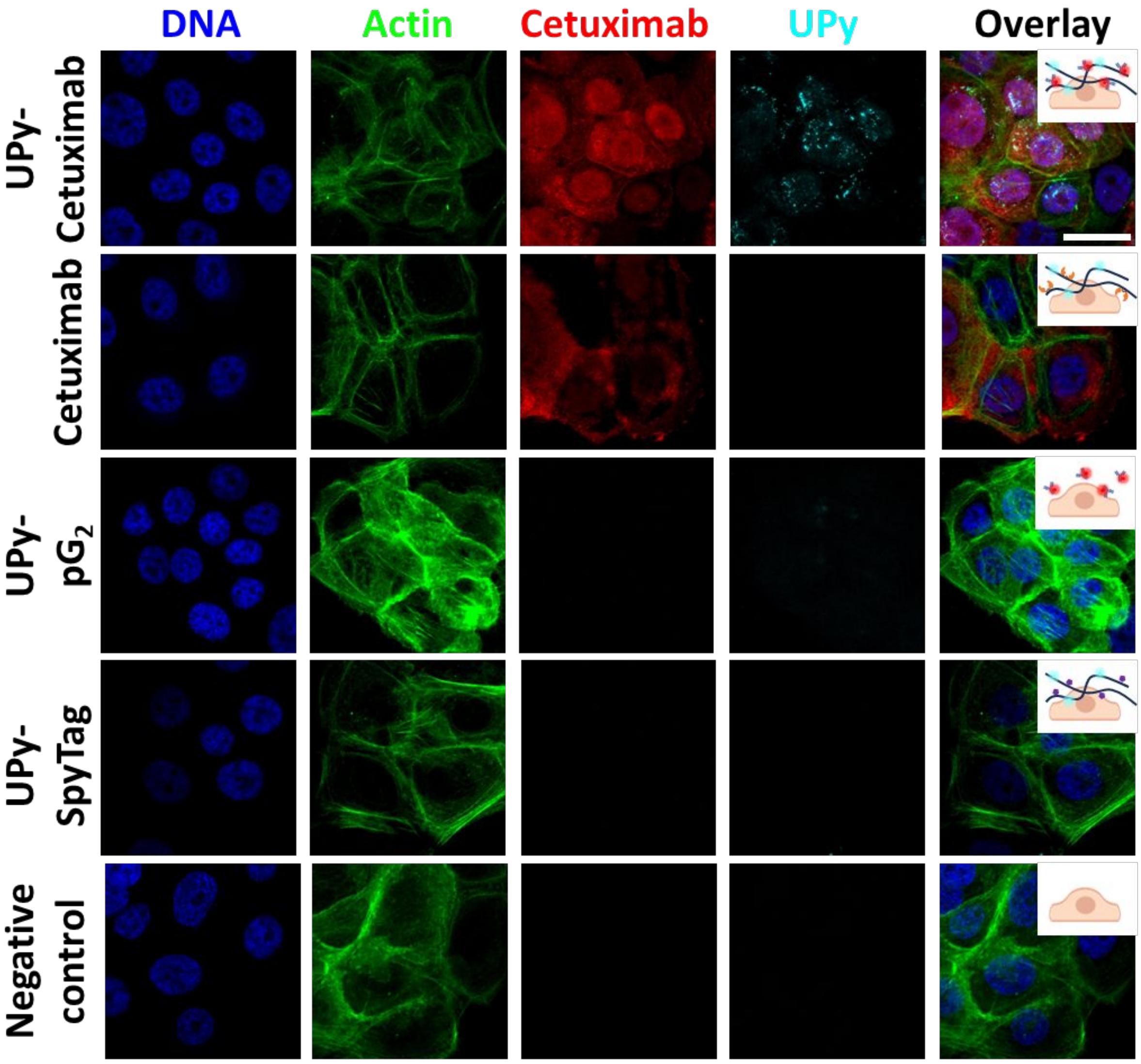


**Fig. S4** | Confocal images of control experiments of cell studies with BxPC-3 cells (UPy-fibers in white, nucleus in blue, actin-cytoskeleton in green, Cetuximab-Alexa647 in red). Antibody only control showing Cetuximab-EGFR binding, UPy-protein G fibers confirming no non-specific binding of UPy-$pG_2$ to BxPC-3 cells, UPy-SpyTag fibers confirming no non-specific binding of UPy-SpyTag to BxPC-3 cells, negative control showing no overlap between fluorescent channels.

## Protein sequences

**mNeonGreen-SpyCatcher003-strep**

mNeonGreen highlighted in green

SpyCatcher003 highlighted in grey

StrepTag II in pink

MGHCMGVSKGEEDNMASLPATHELHIFGSINGVDFDMVGQGTGNPNDGYEELNLKSTKGD
LQFSPWILVPHIGYGFHQYLPYPDGMSPFQAAMVDGSGYQVHRTMQFEDGASLTVNYRYT
YEGSHIKGEAQVKGTGFPADGPVMTNSLTAADWCRSKKTYPNDKTIISTFKWSYTTGNGKR
YRSTARTTYTFAKPMAANYLKNQPMYVFRKTELKHSKTELNFKEWQKAFTDVMGMDELYK
GGSAGGVTTLSGLSGEQGPSGDMTTEEDSATHIKFSKRDEDGRELAGATMELRDSSGKTIS
TWISDGHVKDFYLYPGKYTFVETAAPDGYEVATPIEFTVNEDGQVTVDGEATEGDAHTGGS
GGSGWSHPQFEKGIA*

**strep-pG$_2$-SpyCatcher003-His**

Protein G (pG), including unnatural amino acid, highlighted in yellow

SpyCatcher003 highlighted in grey

6xHis-Tag in red

StrepTag II in pink

MGWSHPQFEKGGSMTFKLIINGKTLKGEITIEAVDA(**Bpa**)EAEKIFKQYANDYGIDGEWTYD
DATKTFTVTEEFTGGSGGSGGSGGSGGSGGSGEFAEAAAKEAAAKEAAAKEAAAKEAAAK
EAAAKAEFGGSGGSGGSGGSGGSGGSGGTMTFKLIINGKTLKGEITIEAVDA(**Bpa**)EAEKIF
KQYANDYGIDGEWTYDDATKTFTVTELTGGSGGSGGSGGSGGSGGSGEFAEAAAKEAAA
KEAAAKEAAAKEAAAKEAAAKAEFGGSGGSGGSGGSGGSGGSGGTGVTTLSGLSGEQGP
SGDMTTEEDSATHIKFSKRDEDGRELAGATMELRDSSGKTISTWISDGHVKDFYLYPGKYTF
VETAAPDGYEVATPIEFTVNEDGQVTVDGEATEGDAHTGSGGSHHHHHH*
